# Application of genetic algorithm combined with improved SEIR model in predicting the epidemic trend of COVID-19, China

**DOI:** 10.1038/s41598-022-12958-z

**Published:** 2022-05-26

**Authors:** Zhenzhen Qiu, Youyi Sun, Xuan He, Jing Wei, Rui Zhou, Jie Bai, Shouying Du

**Affiliations:** School of Chinese Materia Medica, Beijing University of Chinese Medicine, Beijing, 102488 China

**Keywords:** Computational science, Information technology

## Abstract

Since the outbreak of the 2019 Coronavirus disease (COVID-19) at the end of 2019, it has caused great adverse effects on the whole world, and it has been hindering the global economy. It is ergent to establish an infectious disease model for the current COVID-19 epidemic to predict the trend of the epidemic. Based on the SEIR model, the improved SEIR models were established with considering the incubation period, the isolated population, and genetic algorithm (GA) parameter optimization method. The improved SEIR models can predict the trend of the epidemic situation better and obtain the more accurate epidemic-related parameters. Comparing some key parameters, it is capable to evaluate the impact of different epidemic prevention measures and the implementation of different epidemic prevention levels on the COVID-19, which has significant guidance for further epidemic prevention measures.

## Introduction

Since the outbreak of the 2019 Coronavirus disease (COVID-19), it has been threatening people’s lives, livelihoods, economic and social development, etc.^1^. By the end of February 2021, COVID-19 had spread to more than 230 countries, with more than 100 million confirmed cases and 2.5 million deaths^2^. China was the first country to be affected. In the early stage of the COVID-19 outbreak, the Chinese government quickly adopted strict epidemic prevention measures, including lockdown of cities^3^, the establishment of Fangcang shelter hospitals^4^, self-isolation^5^, compulsory isolation^6^ and real-time epidemic tracking^7^, which promptly and effectively contained the large-scale trend of the epidemic in China in a timely manner. However, there are still occasional local small-scale outbreaks of COVID-19. COVID-19 is mainly transmitted by respiratory droplets and contact, but so far there is no cure^8^. Therefore, it is of great significance for future epidemic prevention and control and resource allocation to fit the epidemic situation in many places, to obtain the accurate epidemic model, and to predict the trend of COVID-19.

At the beginning of the twentieth century, Kermack and McKendrick^9^ established the SIR (Susceptible Infected Recovered) model using the nonlinear dynamics method, and began to have the establishment of the dynamic model of infectious diseases and the analysis and research of its dynamics. Then according to the transmission characteristics of different infectious diseases, people developed the SIRS model that considers the secondary disease. Further, the SEIR model that considers the incubation period of the disease was improved. These models opened a new chapter for the study of infectious disease dynamics, and greatly promoted the research progress of infectious diseases^10,11^. In addition, applying other algorithms to the prediction and modeling of infectious disease dynamics can optimize parameters and fit the real infectious disease propagation process better^12,13^. For example, Godio et al.^14^ used a particle swarm optimization (PSO) solver to apply stochastic methods to fit epidemic data to improve the accuracy of SEIR model prediction. With the occurrence of the Novel Coronavirus Pneumonia epidemic, Rezapour et al.^15^ used the Caputo fractional derivative combined with the SEIR mathematical model to predict the spread of COVID-19 in Iran and the world; Marinca et al.^16^ used the optimal auxiliary function method (OAFM) to obtain an approximate solution of the COVID-19 SEIR model, and developed a SEIR model suitable for the number of deaths from COVID-19. Although these models can predict the popular trend of COVID-19 to some extent, there are still some shortages. For example, most scholars do not pre-process the existing data before prediction and analysis, and the prediction accuracy of the models needs to be improved. Compared with other infectious diseases, although COVID-19 has a lower mortality rate, it is more infectious and has a certain infectivity during the incubation period. Besides, objective factors such as isolation measures have a great impact on it, which leads to the inaccuracy of the existing infectious disease prediction model to obtain the true epidemic parameters^17–20^.

**Figure 1:**
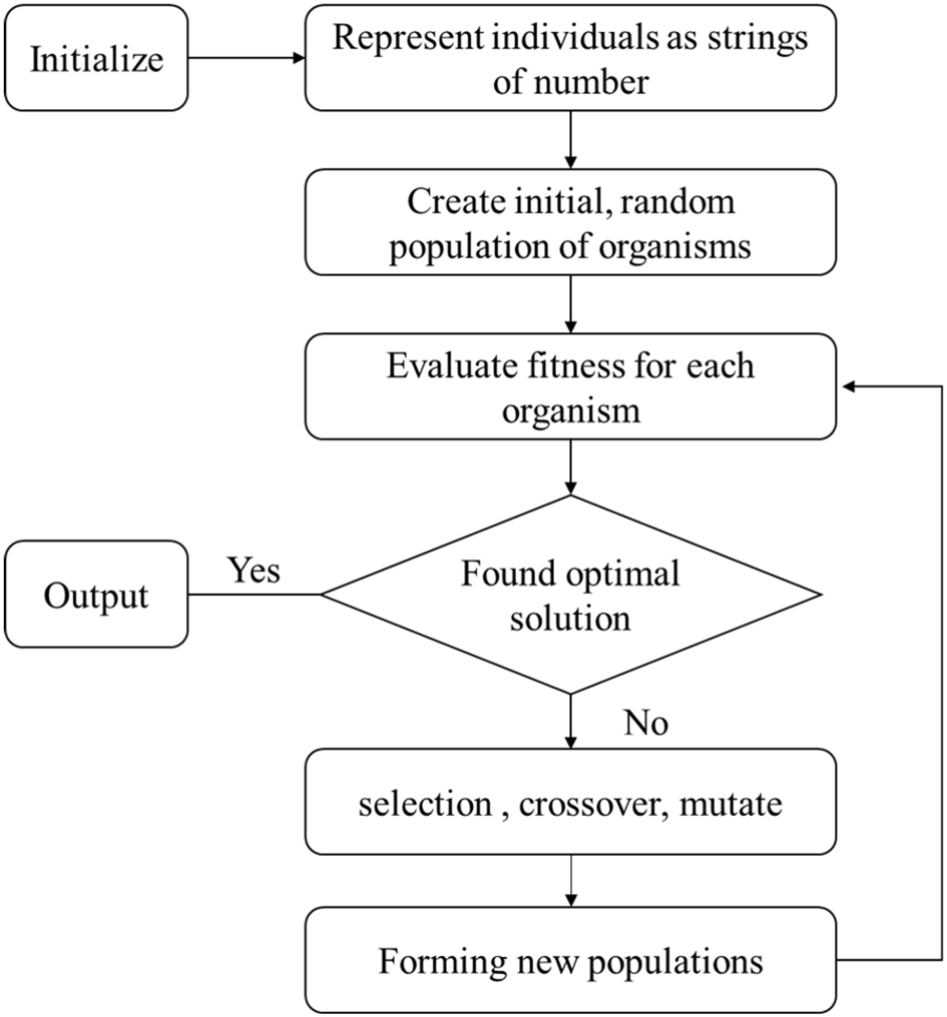
Flow chart of Genetic Algorithm.

Genetic Algorithm (GA) was first proposed by Professor John Holland^21^. It is a method to find the optimal solution or approximate optimal solution to a complex problem by simulating the natural evolution process. It has been used in neural networks^22^, combinatorial optimization^23^, artificial intelligence^24,25^, genetic programming^26^, data mining^27^ and other fields. The optimization principle is shown in Fig. 1. When usually using GA to optimize parameters, we need to set each parameter that needs to be estimated as an individual and set the possible value range of each parameter in the application. GA can encode all parameters to form the initial population, and define the fitness function at the same time. Generally, the root mean square error (RMSE) between the test results and the real results after the parameters are substituted is the standard to measure the quality of the individual. The smaller the error, the better the performance of the population. The optimal solution can be obtained. Finally, the optimal parameter population is assigned to the target model to obtain the final results.

Compared with the traditional optimization algorithm, GA utilizes probabilistic rules instead of certain rule. Therefore, GA has the characteristics of global optimization, simple operation, and it is suitable for solving complex optimization problems. In this research, we used GA to analyze the influence of social and government behaviors on disease dynamics to optimize the model parameters, and took into account the changes of objective factors such as the gradual improvement of isolation measures in the early and late stages of the epidemic. Using simulation software Matlab, two improved SEIR epidemic prediction models were constructed, including the SEIR model. One is considering incubation period infectivity, and the other is considering both incubation period infectivity and isolation measures. The trend of epidemic in different periods was simulated and the prediction results were obtained. The results provide reference for the parameters of epidemic prediction and the improvement and optimization of epidemic prevention measures in the future.

## Methods

### Traditional SEIR infectious disease prediction model

Because COVID-19 has an incubation period, and its average incubation period is 7 days, we chose to consider the SEIR infectious disease model of the latent person as the basis for modeling. In the traditional SEIR model, the population is divided into the following four categories^28^:

Susceptible (S), healthy people who may be infected.

Exposed (E), people who have been infected and have not shown pathological features.

Infected (I), people who have been infected and show pathological features.

Removed (R), people who have died or cured that are no longer contagious and will not be infected.

At the same time, the following assumptions are often made^29,30^:

1. It is assumed that the total population in a certain area is constant, and the natural birth rate and natural death rate are not considered, and the movement of people between regions is not considered.
2. Recovered persons can develop antibodies and will not be infected again recently.
3. The exposed (E) is not contagious.

**Figure 2:**
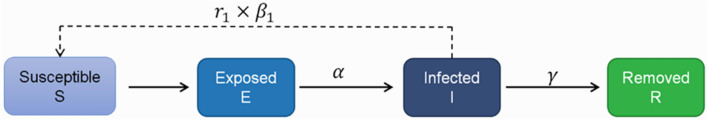
Schematic diagram of the traditional SEIR model.

Therefore, based on the traditional SEIR model, the mutual conversion relationship among the four populations is shown in Fig. 2. The susceptible have a certain probability of being infected into the exposed after contact with the infected, and exposed persons will be transformed into infected persons after a period of incubation period, and the infected persons will be cured or die and become removers. r_1_ is the number of effective contacts of infected persons, and $\beta_{1}$ is the probability of infection of infected persons each time they come into contact with susceptible persons. ${\upalpha }$ is the conversion rate of exposed to infected persons, and ${\upgamma }$ is the removal rate of infected persons, which is the reciprocal of the treatment cycle. At the same time, set the total population in the area to N, and S + E + I + R = N. Establishing differential equations for the above relationship can be obtained:

1 $$\left\{ {\begin{array}{*{20}l} {\frac{{{\text{dS}}}}{{{\text{dt}}}} = - {\text{r}}_{1} {\upbeta }_{1} {\text{I}}\frac{{\text{S}}}{{\text{N}}}} \\ {\frac{{{\text{dE}}}}{{{\text{dt}}}} = {\text{r}}_{1} {\upbeta }_{1} {\text{I}}\frac{{\text{S}}}{{\text{N}}} - {\alpha E}} \\ {\frac{{{\text{dI}}}}{{{\text{dt}}}} = {\alpha E} - {\gamma I}} \\ {\frac{{{\text{dR}}}}{{{\text{dt}}}} = {\gamma I}} \\ \end{array} } \right.$$

By (1) is modified to iterative forms available:

2 $$\left\{ {\begin{array}{*{20}l} {{\text{S}}_{{{\text{t}} + 1}} = {\text{S}}_{{\text{t}}} - {\text{r}}_{1} {\upbeta }_{1} {\text{I}}_{{\text{t}}} \frac{{{\text{S}}_{{\text{t}}} }}{{\text{N}}}} \\ {{\text{E}}_{{{\text{t}} + 1}} = {\text{E}}_{{\text{t}}} + {\text{r}}_{1} {\upbeta }_{1} {\text{I}}_{{\text{t}}} \frac{{{\text{S}}_{{\text{t}}} }}{{\text{N}}} - {\alpha E}_{{\text{t}}} } \\ {{\text{I}}_{{{\text{t}} + 1}} = {\text{I}}_{{\text{t}}} + {\alpha E}_{{\text{t}}} - {\gamma I}_{{\text{t}}} } \\ {{\text{R}}_{{{\text{t}} + 1}} = {\text{R}}_{{\text{t}}} + {\gamma I}_{{\text{t}}} } \\ \end{array} } \right.$$

### Improved SEIR model A: infectious disease prediction model considering the infectivity of incubation period

**Figure 3:**
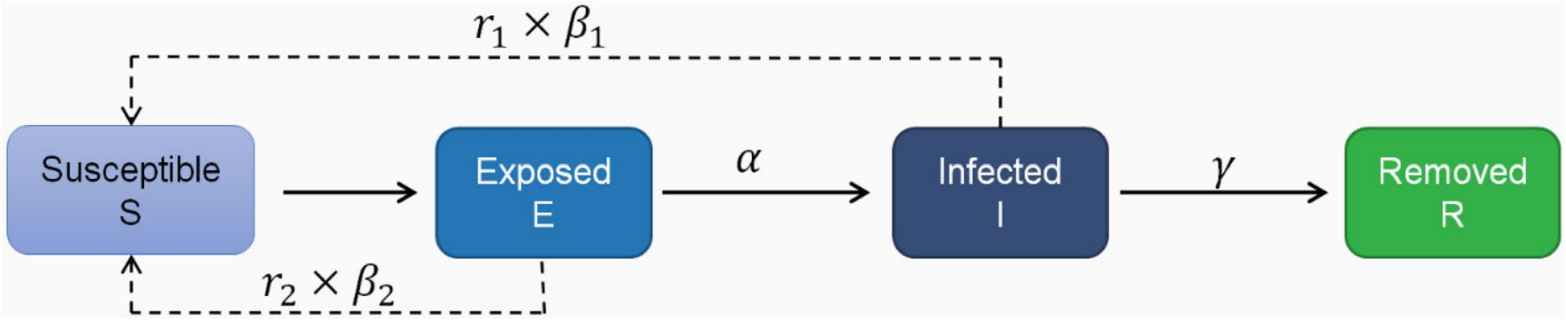
Schematic diagram of SEIR model considering latent infectivity.

According to the “COVID-19 Diagnosis and Treatment Program (Trial Eighth Edition)” issued by the National Health Commission on August 18, 2020, the source of infection is not only the patients infected by novel coronavirus, but also the asymptomatic infection. Namely, COVID-19 is infectious in the incubation period. Therefore, the traditional SEIR model needs to be changed as follows:

As shown in Fig. 3, based on the traditional SEIR model, the infectivity of the latent person to the susceptible person is increased, and the susceptible person may be infected and become a new latent person after contact with the exposed or the infected. Among them, r_2_ is the number of effective contacts of the latent, and ${\upbeta }_{{2}}$ is the infection probability of each contact of the latent with a susceptible person, which can be concluded that the new differential equation:

3 $$\left\{ {\begin{array}{*{20}l} {\frac{{{\text{dS}}}}{{{\text{dt}}}} = - {\text{r}}_{1} {\upbeta }_{1} {\text{I}}\frac{{\text{S}}}{{\text{N}}} - {\text{r}}_{2} {\upbeta }_{2} {\text{E}}\frac{{\text{S}}}{{\text{N}}}} \\ {\frac{{{\text{dE}}}}{{{\text{dt}}}} = {\text{r}}_{1} {\upbeta }_{1} {\text{I}}\frac{{\text{S}}}{{\text{N}}} + {\text{r}}_{2} {\upbeta }_{2} {\text{E}}\frac{{\text{S}}}{{\text{N}}} - {\alpha E}} \\ {\frac{{{\text{dI}}}}{{{\text{dt}}}} = {\alpha E} - {\gamma I}} \\ {\frac{{{\text{dR}}}}{{{\text{dt}}}} = {\gamma I}} \\ \end{array} } \right.$$

By (3) is modified to iterative forms available:

4 $$\left\{ {\begin{array}{*{20}l} {{\text{S}}_{{{\text{t}} + 1}} = {\text{S}}_{{\text{t}}} - {\text{r}}_{1} {\upbeta }_{1} {\text{I}}\frac{{{\text{S}}_{t} }}{{\text{N}}} - {\text{r}}_{2} {\upbeta }_{2} {\text{E}}\frac{{{\text{S}}_{t} }}{{\text{N}}}} \\ {{\text{E}}_{t + 1} = {\text{E}}_{t} + {\text{r}}_{1} {\upbeta }_{1} {\text{I}}_{t} \frac{{{\text{S}}_{t} }}{{\text{N}}} + {\text{r}}_{2} {\upbeta }_{2} {\text{E}}_{t} \frac{{{\text{S}}_{t} }}{{\text{N}}} - {\alpha E}_{t} } \\ {{\text{I}}_{t + 1} = {\text{I}}_{t} + {\alpha E}_{t} - {\gamma I}_{t} } \\ {{\text{R}}_{t + 1} = {\text{R}}_{t} + {\gamma I}_{t} } \\ \end{array} } \right.$$

### Improved SEIR model B: predicting infectious diseases considering the infectivity of incubation period and isolation measures

**Figure 4:**
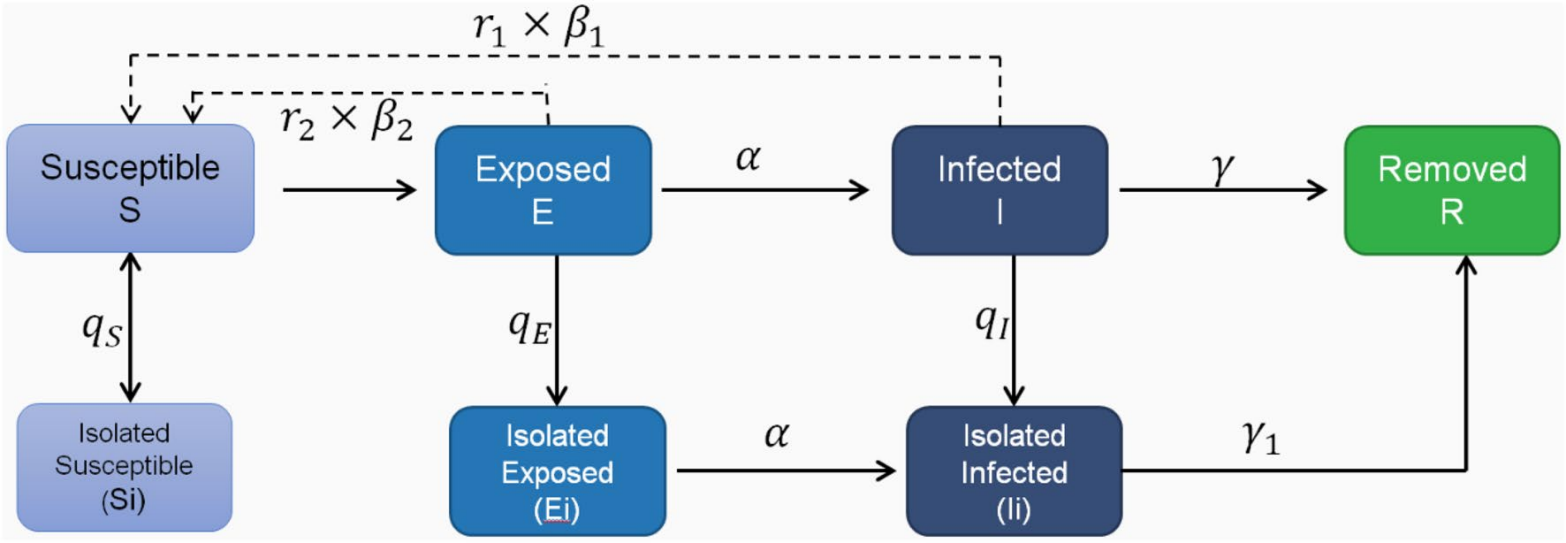
Schematic diagram of SEIR model considering incubation infectivity and isolation measures.

In the early stage of the epidemic, due to the active actions of the government, many isolation measures were taken, such as the isolation treatment of infected people in Fangcang shelter hospitals, the centralized observation and home isolation. Isolation treatments are depending on the close contacts of COVID-19. The policy provides for different levels of isolation of the susceptible, the exposed and the infected, which plays a key role in the trend of the epidemic situation. Based on the existing epidemic prevention and control measures in China, the SEIR model B was further improved in order to simulate the trend of the epidemic accurately:

As shown in Fig. 4, on the basis of the previous model, measures to isolate various groups of people are considered. First infected after diagnosis will be quarantined as isolators ${\text{I}}_{g}$ no longer contagious infection^31^, and after exposed (E), infected (I) contact with susceptible people (S), all in close contact with infected people (I) became the exposed (E), while the uninfected close contacts remain in the susceptible crowd (S). The exposed (E) will be quarantined after the nucleic acid test result is positive, which is called exposed isolator ${\text{E}}_{g}$. The relevant uninfected contacts will be isolated as susceptible isolators ${\text{S}}_{g}$. Among them, ${\text{S}}_{g}$ will not be infected during the isolation period, and will return to susceptible crowd (S) after the end of the isolation period (set the isolation period as ${\upmu }$). Susceptible crowd (S) have the possibility of being infected again. After the incubation period is over, if the exposed (E) who have been the isolated will be confirmed cases of COVID-19 and become infected isolator ${\text{I}}_{g}$, ${\text{E}}_{g}$ and ${\text{I}}_{g}$ are no longer infectious because they are isolated. In the end, the infected and infected isolators will be cured or die as the remover. Where, ${\text{q}}_{S}$ is the proportion of close contacts isolated among the susceptible, ${\text{q}}_{E}$ is the probability of the exposed being isolated, and ${\text{q}}_{I}$ is the probability of the infected being isolated. Based on the above population relations, the original differential equation can be extended as follows:

5 $$\left\{ {\begin{array}{*{20}l} {\frac{dS}{{dt}} = - \frac{{S\left[ {r_{1} I\left( {\beta_{1} + q_{S} - q_{S} \beta_{1} } \right) + r_{2} E\left( {\beta_{2} + q_{S} - q_{S} \beta_{2} } \right)} \right]}}{{N - S_{g} - E_{g} - I_{g} }}} \\ {\frac{{dS_{g} }}{dt} = \frac{{Sq_{S} \left[ {r_{1} I\left( {1 - \beta_{1} } \right) + r_{2} E\left( {1 - \beta_{2} } \right)} \right]}}{{N - S_{g} - E_{g} - I_{g} }}} \\ {\frac{dE}{{dt}} = \frac{{S\left( {r_{1} \beta_{1} I + r_{2} \beta_{2} E} \right)}}{{N - S_{g} - E_{g} - I_{g} }} - \alpha E\left( {1 - q_{E} } \right) - Eq_{E} } \\ \begin{gathered} \frac{dEg}{{dt}} = Eq_{E} - \alpha E_{g} \hfill \\ \frac{dI}{{dt}} = \alpha E\left( {1 - q_{E} } \right) - Iq_{I} - I\gamma \left( {1 - q_{I} } \right) \hfill \\ \frac{{dI_{g} }}{dt} = E_{g} \alpha + Iq_{I} - I_{g} \gamma_{1} \hfill \\ \frac{dR}{{dt}} = I\gamma \left( {1 - q_{I} } \right) + I_{g} \gamma_{1} \hfill \\ \end{gathered} \\ \end{array} } \right.$$

For the convenience of calculation, let $\frac{dA}{{dt}} = \frac{{\text{S}}}{{{\text{N}} - {\text{S}}_{{\text{g}}} - {\text{E}}_{{\text{g}}} - {\text{I}}_{{\text{g}}} }}$, in (5) and modified to iterative forms available:

6 $$\left\{ {\begin{array}{*{20}l} {{\text{A}}_{{\text{t}}} = \frac{{{\text{S}}_{{\text{t}}} }}{{{\text{N}} - {\text{S}}_{{{\text{gt}}}} - {\text{E}}_{{{\text{gt}}}} - {\text{I}}_{{{\text{gt}}}} }}} \\ {{\text{S}}_{{{\text{t}} + 1}} = {\text{S}}_{{\text{t}}} - {\text{A}}_{{\text{t}}} {\text{r}}_{1} {\text{I}}_{{\text{t}}} \left( {{\upbeta }_{1} + {\text{q}}_{{\text{S}}} - {\text{q}}_{{\text{S}}} {\upbeta }_{1} } \right) - {\text{A}}_{{\text{t}}} {\text{r}}_{2} {\text{E}}_{{\text{t}}} \left( {{\upbeta }_{2} + {\text{q}}_{{\text{S}}} - {\text{q}}_{{\text{S}}} {\upbeta }_{2} } \right)} + {\text{S}}_{{{\text{g}}\left( {{\text{t}} - {\upmu }} \right)}} \\ {{\text{S}}_{{{\text{g}}\left( {{\text{t}} + 1} \right)}} = {\text{S}}_{{{\text{gt}}}} + {\text{A}}_{{\text{t}}} {\text{q}}_{{\text{S}}} \left[ {{\text{r}}_{1} {\text{I}}_{{\text{t}}} \left( {1 - {\upbeta }_{1} } \right) + {\text{r}}_{2} {\text{E}}_{{\text{t}}} \left( {1 - {\upbeta }_{2} } \right)} \right] - {\text{S}}_{{{\text{g}}\left( {{\text{t}} - {\upmu }} \right)}} } \\ \begin{gathered} {\text{E}}_{{{\text{t}} + 1}} = {\text{E}}_{{\text{t}}} + {\text{A}}_{{\text{t}}} \left( {{\text{r}}_{1} {\upbeta }_{1} {\text{I}}_{{\text{t}}} + {\text{r}}_{2} {\upbeta }_{2} {\text{E}}_{{\text{t}}} } \right) - {\alpha E}_{{\text{t}}} \left( {1 - {\text{q}}_{{\text{E}}} } \right) - {\text{E}}_{{\text{t}}} {\text{q}}_{{\text{E}}} \hfill \\ {\text{E}}_{{{\text{g}}\left( {{\text{t}} + 1} \right)}} = {\text{E}}_{{{\text{gt}}}} + {\text{E}}_{{\text{t}}} {\text{q}}_{{\text{E}}} - {\alpha E}_{{{\text{gt}}}} \hfill \\ {\text{I}}_{{{\text{t}} + 1}} = {\text{I}}_{{\text{t}}} + {\text{E}}_{{\text{t}}} {\upalpha }\left( {1 - {\text{q}}_{{\text{E}}} } \right) - {\text{I}}_{{\text{t}}} {\text{q}}_{{\text{I}}} - {\text{I}}_{{\text{t}}} {\upgamma }\left( {1 - {\text{q}}_{{\text{I}}} } \right) \hfill \\ {\text{I}}_{{{\text{g}}\left( {{\text{t}} + 1} \right)}} = {\text{I}}_{{{\text{gt}}}} + {\text{E}}_{{{\text{gt}}}} {\upalpha } + {\text{I}}_{{\text{t}}} {\text{q}}_{{\text{I}}} - {\text{I}}_{{{\text{gt}}}} {\upgamma }_{1} \hfill \\ {\text{R}}_{{{\text{t}} + 1}} = {\text{R}}_{{\text{t}}} + {\text{I}}_{{\text{t}}} {\upgamma }\left( {1 - {\text{q}}_{{\text{I}}} } \right) + {\text{I}}_{{{\text{gt}}}} {\upgamma }_{1} \hfill \\ \end{gathered} \\ \end{array} } \right.$$

### Parameter estimation and model fitting

The results of improved SEIR models, A and B, are greatly affected by the initial parameters. In order to establish the SEIR infectious disease model, appropriate values of the key parameters should be selected, which are E, ${\text{q}}_{S}$, ${\text{q}}_{E}$, ${\text{q}}_{I}$, ${\text{r}}_{1}$, ${\upbeta }_{1}$, ${\text{r}}_{2}$, ${\upbeta }_{2}$, ${\upalpha }$, ${\upgamma }$, ${\upgamma }_{1}$, ${\upmu }$. Among them, the incidence probability (${\upalpha }$) of the exposed is taken as the inverse of the incubation period, and the incubation period is taken as 7 days in line with most reports^32,33^, namely ${\upalpha } = \frac{1}{7} \approx 0.1429$. According to the isolation policy of Wuhan and Beijing, the isolution period is 14 days, namely μ = 14. ${\text{q}}_{E}$ is the isolation rate of the exposed and ${\text{q}}_{I}$ is the isolation probability of the infected. According to the current epidemic prevention and control strategy, all confirmed patients will be isolated, so ${\text{q}}_{E}$ is the accuracy rate of confirmed patients and ${\text{q}}_{{\text{I}}} = {1}$. According to a report on April 18, 2020, the accuracy of nucleic acid detection is about 50% to 70%, and with the epidemic under control, the number of existing cases is decreasing, and nucleic acid kits are sufficient. The accuracy rate should be improved compared with the initial stage of the epidemic, so its maximum value is set, namely ${\text{q}}_{E}$ = 0.7. Other parameters are estimated independently according to different conditions in different regions.

## Result

### Predicting the trend of COVID-19 in Wuhan with the improved SEIR models

According to the above mentioned improved SEIR models and GA, the epidemic situation in Wuhan was simulated. Wuhan epidemic is the earliest COVID-19 epidemic in China. Due to insufficient nucleic acid detection kits in the early stage and insufficient cognition of COVID-19, there may be some deviation between the earlier official reported data and the actual data^34,35^. Wuhan has been closed since January 23, 2020. In order to ensure reliable data, we selected the epidemic related data of January 25, 2020 solstice and March 20 in Wuhan for collation and simulation. According to the dynamics of the epidemic situation in Wuhan, piecewise function can be used for modeling^36^. According to the first report of Wuhan Health Commission on February 8, 2020, the number of people receiving isolation treatment was divided into sections. During the period of January 25th and February 7th, Fangcang shelter hospitals, Huo shenshan hospital and Lei shenshan hospital under construction were not fully put into use, and the medical resources were relatively limited and could not provide perfect isolation measures. Therefore, the model in Eq. (4), which only considered the infectivity in the incubation period but did not consider the isolation measures, was used for data simulating. During the period of 8 February and 10 March, the isolation measures were guaranteed as the isolation hospitals were put into operation. Therefore, the improved SEIR model in Eq. (6) considering the infectivity of incubation period and isolation measures was used for data simulation. The data of epidemic in Wuhan was taken as the data collection area. Due to the strict city closure measures adopted by Wuhan since January 23, inter-regional personnel flow was not considered. The total population in Wuhan is set as *N* = 11,081,000 according to Wuhan Statistical Yearbook 2019 released by Wuhan Statistical Bureau^37^. The remaining unknown parameters were optimized by GA, and the RMSE of I and R were used as the fitness function values to estimate the parameters. Table 1 shows the estimated results of various parameters of the epidemic in Wuhan.

**Table 1:** Estimated results of epidemic parameters in Wuhan.

Parameters	${\text{r}}_{1}$	${\text{r}}_{2}$	$\beta_{1}$	$\beta_{2}$	${\upgamma }$	${\upgamma }_{1}$	${\text{q}}_{S}$	E
Assignment	4.6701	5.1396	0.4896	0.0244	0.7401	0.0631	0.0046	606

**Figure 5:**
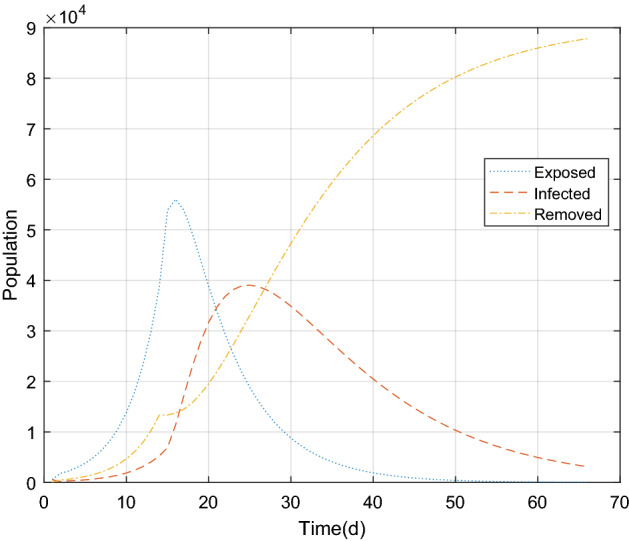
The population of exposed, infected and removed in Wuhan over time.

**Figure 6:**
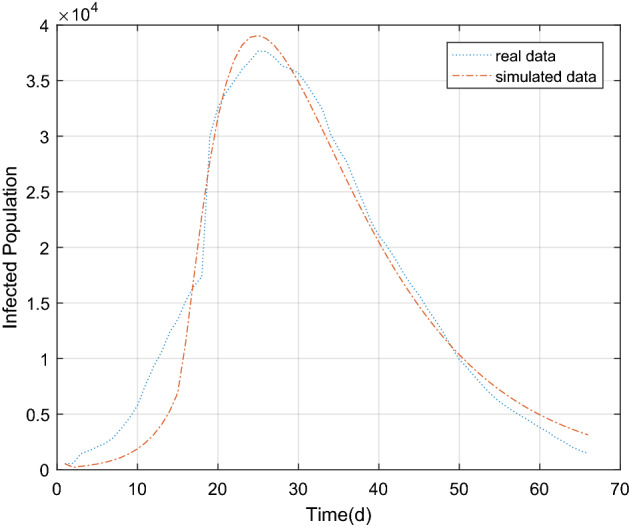
Comparison between simulated data and real data of the existing infected in Wuhan.

The parameter estimation results were substituted into the piecewide differential equation to obtain the fitting results of the epidemic in Wuhan, as shown in Figs. 5 and 6 below. There was a good correlation between the model simulating results and the real value (r = 0.9849, *P* < 0.05). It is proved that the combination of GA and improved SEIR models can fit the existing epidemic data better and restore the unknown parameters.

### Predicting the trend of COVID-19 in Beijing with the improved SEIR model

In the same method, we sumulating the epidemic outbreak of COVID-19 in Beijing Xinfadi. Unlike the initial outbreak in Wuhan, a complete epidemic prevention and control system had been established in Beijing at the first time of the outbreak in Xinfadi. Therefore, the improved SEIR model B for predicting the trend of COVID-19 was used for simulation. The premise is to take Beijing as a fixed region, without considering inter-regional mobility. According to the 2019 Beijing Statistical Yearbook released by the Beijing Municipal Bureau of Statistics, the total population of Beijing is set as *N* = 21,536,000^38^. The remaining unknown parameters were optimized by GA, and the RMSE of I and R was used as the fitness function values to estimate the parameters. Table 2 shows the estimated results of the epidemic parameters in Beijing Xinfadi.

**Table 2:** Estimated results of epidemic parameters in Beijing Xinfadi.

Parameters	${\text{r}}_{1}$	${\text{r}}_{2}$	$\beta_{1}$	$\beta_{2}$	${\upgamma }$	${\upgamma }_{1}$	${\text{q}}_{S}$	E
Assignment	1.373	1.676	0.959	0.350	0.901	0.114	0.007	150

**Figure 7:**
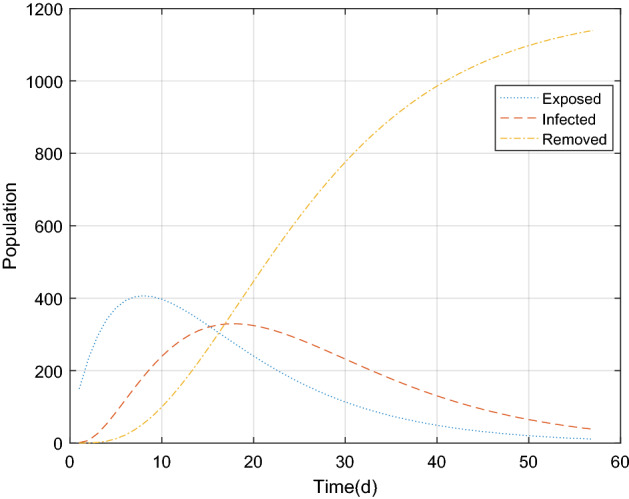
The population of exposed, infected and removed in Beijing Xinfadi over time.

**Figure 8:**
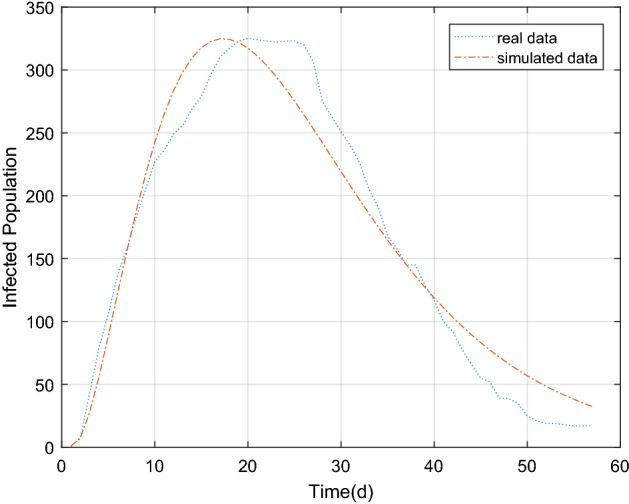
Comparison between simulated data and real data of the existing infected in Beijing Xinfadi.

The estimated parameters in Table 2 were substituted into the differential equation in Eq. (6) to obtain the simulating results of the epidemic situation in Beijing Xinfadi, as shown in Figs. 7 and 8 below. Based on the comparison of the estimated parameters between Wuhan and Beijing, it can be seen that with the trend of the epidemic situation, the parameters will vary greatly. Comparsion with ${\text{r}}_{1}$ and ${\text{r}}_{2}$, it can be seen that differences on the initial stage of the epidemic, individual protection has been improved, and the effective contact rate of the exposed (E) and the infected (I) is still reduced even after the resumption of work and production. At the same time, the comparison of ${\upgamma }_{1}$ and ${\upgamma }$ shows that because the number of COVID-19 cases in Beijing Xinfadi is far less than that in Wuhan and the medical resources are abundant, the cure rate of COVID-19 in Beijing Xinfadi is greatly improved.

## Discussion

On the one hand, the parameters optimized by GA can intuitively express the effectiveness of various epidemic prevention measures in different regions at different times, which provide reference for epidemic prediction in the future. On the other hand, compared with the traditional SEIR model, the improved SEIR model considered the latent infectivity and human intervention measures to establish differential equations including the new population and the original four groups of people, so as to show the influence of the latent infectivity and human intervention on the trend of COVID-19 in the process of epidemic transmission. Therefore, the use of the improved SEIR model can fit the trend of COVID-19 epidemic better. Meanwhile, epidemic prevention measures have great impact on epidemic parameters. Then the improved SEIR model can quickly analyze the effectiveness of existing epidemic prevention measures.

According to the estimated epidemic parameters in Wuhan and Beijing Xinfadi, the severity of the epidemic and the prevention and control degree in different places at different times can be compared. The values of ${\text{r}}_{1}$ and ${\text{r}}_{2}$ are influenced by individual protection, such as wearing masks and disinfection frequency, as well as policy regulation, such as entertainment places where people gather are not open to the public, home isolation, and crowd control in indoor places^39,40^. From the results of the numerical comparison, it can be seen that the ${\text{r}}_{1}$ and ${\text{r}}_{2}$ values of the Wuhan epidemic are much larger than the values of the Beijing Xinfadi epidemic. This may be due to the shortage of masks and protective clothing and other materials in the early stage of the Wuhan epidemic, and the lack of awareness of the people to wear masks correctly, which caused the people to be unable to achieve effective individual protection. With the improvement of prevention and control policies and the popularization of epidemic prevention knowledge, it can be seen that in the half a year(approx. 2020.7–2020.12), ${\text{r}}_{1}$ and ${\text{r}}_{2}$ decreased significantly in Beijing Xinfadi.

The simulation results of existing cases in Wuhan and Beijing Xinfadi showed a good correlation with the true values (Wuhan: r = 0.9849, *P* < 0.05; Beijing Xinfadi: r = 0.9764, *P* < 0.05). As can be seen from the Figs. 6 and 8, the simulation results of COVID-19 in Wuhan were even better. The simulation results at the initial stage of the epidemic are different from the true value of the trend of the epidemic, which may be due to the lack of nucleic acid testing capabilities in Wuhan at the initial stage, and the number of confirmed and tested daily is related to the amount of testing. It is also possible that the simulation results of the peak of the epidemic case are higher than the true value because the initial detection ability is insufficient. What’s more, some infected (I) are not diagnosed, and this error has to be accepted. Compared with the simulating results in Wuhan, the simulating results in Beijing are not very ideal. The reason is that a small cluster of cases was generated in the early stage of the new outbreak, and the effectiveness of the epidemic prevention and control policy cannot be reflected in the simulation. After the first batch of clustered cases were isolated and treated, the epidemic was effectively controlled. The strict isolation measures are effective, resulting in deviation of simulation results from the real epidemic data. In order to improve the accuracy of the simulating, piecewise simulating can be performed with the time points of occurrence and elimination. In fact, the piecewise function function that was used to predict the trend of COVID-19 in Wuhan performed greatly.

## Conclusion

In this research, GA as a common optimization algorithm is appropriate for estimating unknown parameters in the improved SEIR models A and B, and the simulated parameters can also be used to predict unknown epidemic trends. Compared with the short-term prediction of the number of infected cases of the ANFIS model, GA combined with the improved SEIR models in this study were able to predict the entire epidemic period better. It can be seen from the comparison diagrams that the overall trend from the initial stage, peak, turning point and zero clearance of the COVID-19 epidemic is accurate (r = 0.9849). In addition, compared with Caputo derivative of fractional order, using GA to estimate the optimal values is very simple. This method in our research is also considering the following impact factors, such as different prevention and control strategies to evaluate the impact of different prevention measures and levels on COVID-19. Based on our research, the improved SEIR models can accurately predict epidemic trend in epidemic regions and countries. Because the real data is affected by many aspects, piecewise function performed greatly and can be used for the further optimization. These results show that GA combined with the improved SEIR models are highly adaptable and suitable for the prediction of the trend of COVID-19. The estimation of propagation parameters plays an important role in inflection points and peak prediction, epidemic trend calculations, and simulated transmission dynamics. This research will provide a certain reference for the advance deployment of medical resources during the epidemic and the arrangements for resuming work and production after the epidemic (Supplementary Information).

## Supplementary Information

Supplementary Information 1.

Supplementary Information 2.

## Data Availability

Corresponding databases were established based on the epidemic data reported by the official website of Wuhan Municipal Health Commission (http://wjw.wuhan.gov.cn/) from June 11 to August 6, 2020 and the official website of Beijing Municipal Health Commission (http://wjw.beijing.gov.cn/) from June 11 to August 6, 2020.
