# Supplementary material for: Application of genetic algorithm combined with improved SEIR model in predicting the epidemic trend of COVID-19, China
Source: Sci Rep. 2022 May 26;12:8910. doi: 10.1038/s41598-022-12958-z (PMC9133826; doi:10.1038/s41598-022-12958-z)
Supplement: Supplementary file 1 — Supplementary Information 1. [file 41598_2022_12958_MOESM1_ESM.docx]

%SEIR模型

clear;clc;

%% 参数设置

load Ireal

load Rreal

qs=0.007

r1=1.373

r2=1.676

b1=0.959

b2=0.35

y=0.901

y1=0.114

k=0

E=150

N=21536000;%北京人口

I=1;%传染者

R=0;%康复者

Sg=0

Eg=0

Ig=0

S=N-I-E-R;%易感染者

a =0.143;%潜伏者患病概率

qe=0.7 %核酸检测准确性

qi=1

y=y+k %移除率

y1=y1+k

u=14 %隔离期

T=1:57;

A=S/(N-Sg-Eg-Ig)

%% 运算过程

for t =1:length(T)-1

N(t+1)=N(t)

if t <15 %

S(t+1)=S(t)-A(t)*r1*I(t)*(b1+qs-qs*b1)-A(t)*r2*E(t)*(b2+qs-qs*b2);%易感人数迭代

Sg(t+1)=Sg(t)+A(t)*qs*[r1*I(t)*(1-b1)+r2*E(t)*(1-b2)]

E(t+1)=E(t)+A(t)*(r1*b1*I(t)+r2*b2*E(t))-a*E(t)*(1-qe)-E(t)*qe%潜伏者人数迭代

Eg(t+1)=Eg(t)+E(t)*qe-a*Eg(t)

I(t+1)=I(t)+a*E(t)*(1-qe)-I(t)*qi-I(t)*y*(1-qi);%患病人数迭代

Ig(t+1)=Ig(t)+Eg(t)*a+I(t)*qi-Ig(t)*y1

R(t+1)=R(t)+y1*Ig(t)+I(t)*y*(1-qi);%移除人数迭代

else

S(t+1)=S(t)-A(t)*r1*I(t)*(b1+qs-qs*b1)-A(t)*r2*E(t)*(b2+qs-qs*b2)+Sg(t-u);%易感人数迭代

Sg(t+1)=Sg(t)+A(t)*qs*[r1*I(t)*(1-b1)+r2*E(t)*(1-b2)]-Sg(t-u)

E(t+1)=E(t)+A(t)*(r1*b1*I(t)+r2*b2*E(t))-a*E(t)*(1-qe)-E(t)*qe%潜伏者人数迭代

Eg(t+1)=Eg(t)+E(t)*qe-a*Eg(t)

I(t+1)=I(t)+a*E(t)*(1-qe)-I(t)*qi-I(t)*y*(1-qi);%患病人数迭代

Ig(t+1)=Ig(t)+Eg(t)*a+I(t)*qi-Ig(t)*y1

R(t+1)=R(t)+y1*Ig(t)+I(t)*y*(1-qi);%移除人数迭代

end

A(t+1)=S(t+1)/(N(t+1)-Sg(t+1)-Eg(t+1)-Ig(t+1))

end

%% 评价指标计算

Eend=E+Eg

Send=S+Sg

Iend=I+Ig

%% 画图

figure(1)

plot(T,Eend,':',T,Iend,'--',T,R,'-.');

grid on;

xlabel('Time(d)');

ylabel('Population');

legend('Exposed','Infected','Removed');

figure(2)

plot(T,Ireal,':',T,Iend,'-.')

grid on;

xlabel('Time(d)');

ylabel('Infected Population');

legend('real data','simulated data');

rmse1= sqrt(mean((Iend - Ireal).^2))

rmse2= sqrt(mean((R - Rreal).^2))

Ireal=Ireal'

Iend=Iend'

[r,p]=corr(Ireal,Iend,'type','Pearson')
