# Supplementary material for: Application of genetic algorithm combined with improved SEIR model in predicting the epidemic trend of COVID-19, China
Source: Sci Rep. 2022 May 26;12:8910. doi: 10.1038/s41598-022-12958-z (PMC9133826; doi:10.1038/s41598-022-12958-z)
Supplement: Supplementary file 2 — Supplementary Information 2. [file 41598_2022_12958_MOESM2_ESM.docx]

clc,clear

qs=0.0046

r1=4.6701

r2=5.1396

b1=0.4896

b2=0.0244

y=0.7401

y1=0.0631

k=0.0121

E=606

load Ireal_wh

load Rreal_WH

N=11081000;%武汉市人口

I=533;%传染者

R=85;%康复者

Sg=0

Eg=0

Ig=0

S=N-I-E-R;%易感染者

a =0.142;%潜伏者患病概率

qe=0.7 %核酸检测准确性

qi=1

y=y+k %移除率

y1=y1+k

u=14 %隔离期

T=1:66;

A=S/(N-Sg-Eg-Ig)

%% 运算过程

for t =1:length(T)-1

N(t+1)=N(t)

if t <14 % 第14天,2月8号开始隔离

S(t+1)=S(t)-r1*b1*I(t)*S(t)/N(t)-r2*b2*E(t)*S(t)/N(t);%易感人数迭代

E(t+1)=E(t)+r1*b1*S(t)*I(t)/N(t)+r2*b2*E(t)*S(t)/N(t)-a*E(t)%潜伏者人数迭代

I(t+1)=I(t)+a*E(t)-y*I(t);%患病人数迭代

R(t+1)=R(t)+y*I(t);%移除人数迭代

A(t+1)=A(t)

Sg(t+1)=Sg(t)

Eg(t+1)=Eg(t)

Ig(t+1)=Ig(t)

else

if t<28

S(t+1)=S(t)-A(t)*r1*I(t)*(b1+qs-qs*b1)-A(t)*r2*E(t)*(b2+qs-qs*b2);%易感人数迭代

Sg(t+1)=Sg(t)+A(t)*qs*[r1*I(t)*(1-b1)+r2*E(t)*(1-b2)]

E(t+1)=E(t)+A(t)*(r1*b1*I(t)+r2*b2*E(t))-a*E(t)*(1-qe)-E(t)*qe%潜伏者人数迭代

Eg(t+1)=Eg(t)+E(t)*qe-a*Eg(t)

I(t+1)=I(t)+a*E(t)*(1-qe)-I(t)*qi-I(t)*y*(1-qi);%患病人数迭代

Ig(t+1)=Ig(t)+Eg(t)*a+I(t)*qi-Ig(t)*y1

R(t+1)=R(t)+y1*Ig(t)+I(t)*y*(1-qi);%移除人数迭代

else

S(t+1)=S(t)-A(t)*r1*I(t)*(b1+qs-qs*b1)-A(t)*r2*E(t)*(b2+qs-qs*b2)+Sg(t-u);%易感人数迭代

Sg(t+1)=Sg(t)+A(t)*qs*[r1*I(t)*(1-b1)+r2*E(t)*(1-b2)]-Sg(t-u)

E(t+1)=E(t)+A(t)*(r1*b1*I(t)+r2*b2*E(t))-a*E(t)*(1-qe)-E(t)*qe%潜伏者人数迭代

Eg(t+1)=Eg(t)+E(t)*qe-a*Eg(t)

I(t+1)=I(t)+a*E(t)*(1-qe)-I(t)*qi-I(t)*y*(1-qi);%患病人数迭代

Ig(t+1)=Ig(t)+Eg(t)*a+I(t)*qi-Ig(t)*y1

R(t+1)=R(t)+y1*Ig(t)+I(t)*y*(1-qi);%移除人数迭代

end

A(t+1)=S(t+1)/(N(t+1)-Sg(t+1)-Eg(t+1)-Ig(t+1))

end

end

%% 评价指标计算

Eend=E+Eg

Send=S+Sg

Iend=I+Ig

figure(1)

plot(T,Eend,':',T,Iend,'--',T,R,'-.');

grid on;

xlabel('Time(d)');

ylabel('Population');

legend('Exposed','Infected','Removed');

figure(2)

plot(T,Ireal_wh,':',T,Iend,'-.')

grid on;

xlabel('Time(d)');

ylabel('Infected Population');

legend('real data','simulated data');

rmse1= sqrt(mean((Iend-Ireal_wh).^2))

rmse2= sqrt(mean((R-Rreal_WH).^2))

Ireal_wh=Ireal_wh'

Iend=Iend'

[r,p]=corr(Ireal_wh,Iend,'type','Pearson')
